# Supplementary material for: Non-linear association between aspartate aminotransferase to alanine aminotransferase ratio and mortality in critically ill older patients: A retrospective cohort study
Source: PLoS One. 2023 Nov 2;18(11):e0293749. doi: 10.1371/journal.pone.0293749 (PMC10621830; doi:10.1371/journal.pone.0293749)
Supplement: S1 Table — (DOCX) [file pone.0293749.s001.docx]

**S1 Table. Association between AST/ALT ratio and the risk of all-cause mortality in different models using imputed data.**

| **Variable** | **Model 1** | |  | **Model 2** | |  | **Model 3** | |
| --- | --- | --- | --- | --- | --- | --- | --- | --- |
|  | **HR (95% CI)** | ***P* value** |  | **HR (95% CI)** | ***P* value** |  | **HR (95% CI)** | ***P* value** |
| AST/ALT ratio | 1.17 (1.14~1.21) | <0.001 |  | 1.17 (1.13~1.21) | <0.001 |  | 1.04 (1.00~1.07) | 0.040 |
| AST/ALT ratio group |  |  |  |  |  |  |  |  |
| ≤1.24 | Reference |  |  | Reference |  |  | Reference |  |
| 1.25-1.83 | 1.34 (1.20, 1.49) | <0.001 |  | 1.28 (1.15, 1.42) | <0.001 |  | 1.13 (1.02, 1.26) | 0.022 |
| >1.83 | 1.65 (1.49, 1.82) | <0.001 |  | 1.60 (1.45, 1.76) | <0.001 |  | 1.16 (1.05, 1.29) | 0.004 |

**Notes:** Cox proportional hazards regression models were used to calculate hazard ratios (HR) with 95% confidence intervals (CI); Model 1 adjusted for: none; Model 2 adjusted for: age and gender; Model 3 adjusted for: age, gender, smoking, alcoholic, weight, systolic blood pressure, respiratory rate, liver disease, cerebrovascular disease, cardiac arrest, cardiogenic shock, acute kidney injury, sequential organ failure assessment, systemic inflammatory response syndrome, hemoglobin, platelets, anion gap, blood urea nitrogen, and potassium.
