# Supplementary material for: Non-linear association between aspartate aminotransferase to alanine aminotransferase ratio and mortality in critically ill older patients: A retrospective cohort study
Source: PLoS One. 2023 Nov 2;18(11):e0293749. doi: 10.1371/journal.pone.0293749 (PMC10621830; doi:10.1371/journal.pone.0293749)
Supplement: S3 Table — (DOCX) [file pone.0293749.s003.docx]

**S3 Table Threshold effect analysis of AST/ALT ratio on mortality using two-piecewise linear regression with abnormal liver function.**

|  | **HR (95% CI)** | ***P* value** |
| --- | --- | --- |
| Fitting model by standard linear regression | 1.04 (1.01~1.08) | 0.047 |
| Fitting model by two-piece wise linear regression |  |  |
| Inflection point of the AST/ALT ratio | 1.80 |  |
| ≤ 1.80 | 1.51 (1.15~1.99) | < 0.001 |
| > 1.80 | 1.01 (0.96~1.06) | 0.694 |
| *P* for log likelihood ratio test |  | < 0.001 |

**Notes:** We adjusted for: age, gender, smoking, alcoholic, weight, systolic blood pressure, respiratory rate, liver disease, cerebrovascular disease, cardiac arrest, cardiogenic shock, acute kidney injury, sequential organ failure assessment, systemic inflammatory response syndrome, hemoglobin, platelets, anion gap, blood urea nitrogen, and potassium.

**Abbreviations:** HR, hazard ratios; CI, confidence intervals.
