# Supplementary figures and images for: Non-linear association between aspartate aminotransferase to alanine aminotransferase ratio and mortality in critically ill older patients: A retrospective cohort study
Source: PLoS One. 2023 Nov 2;18(11):e0293749. doi: 10.1371/journal.pone.0293749 (PMC10621830; doi:10.1371/journal.pone.0293749)

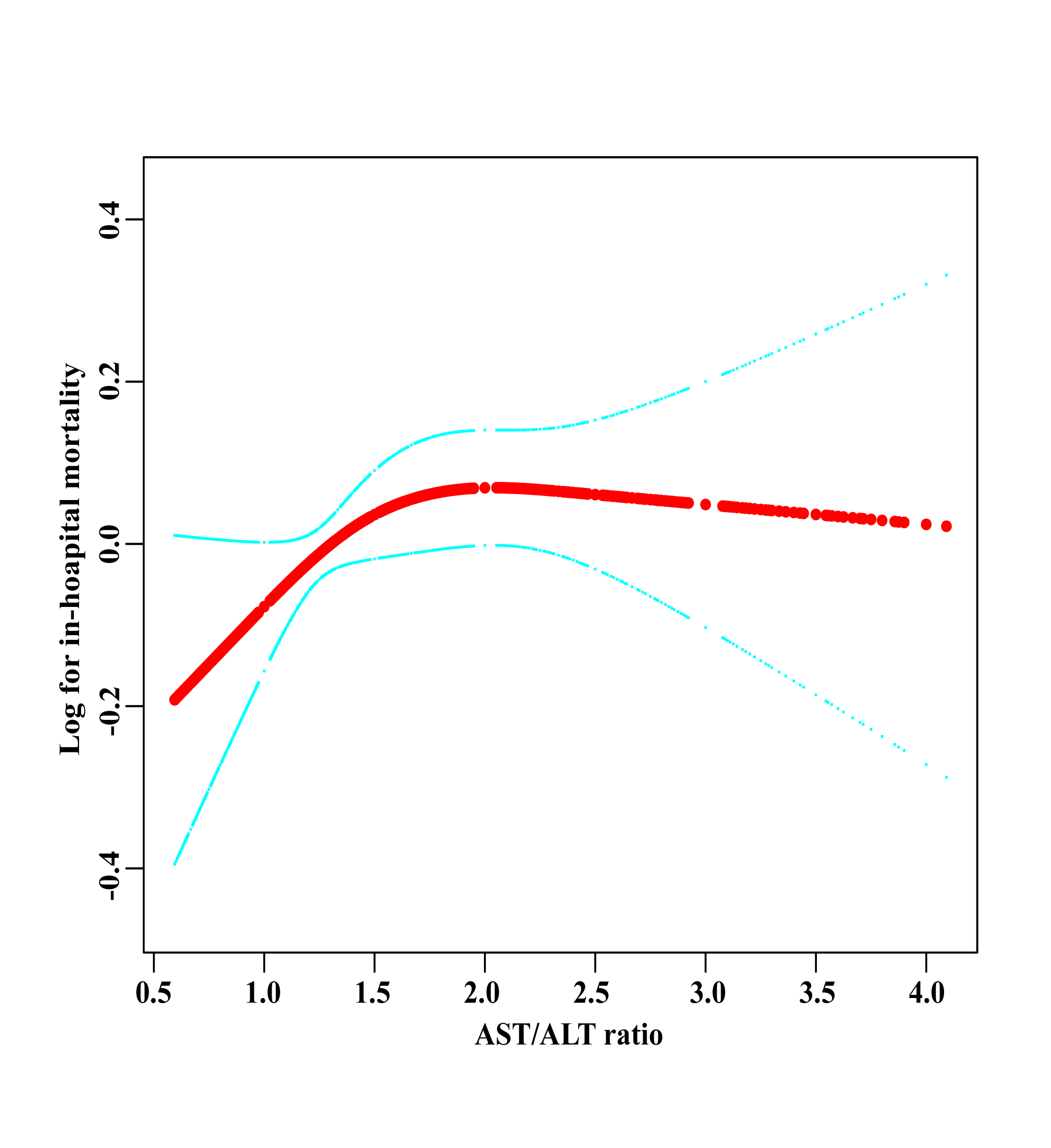

Supplement: S1 Fig — Adjusted for all factors in Model 3. The red lines represent the estimated values, and green lines represent their corresponding 95% confidence intervals. (TIF) [file pone.0293749.s004.tif]

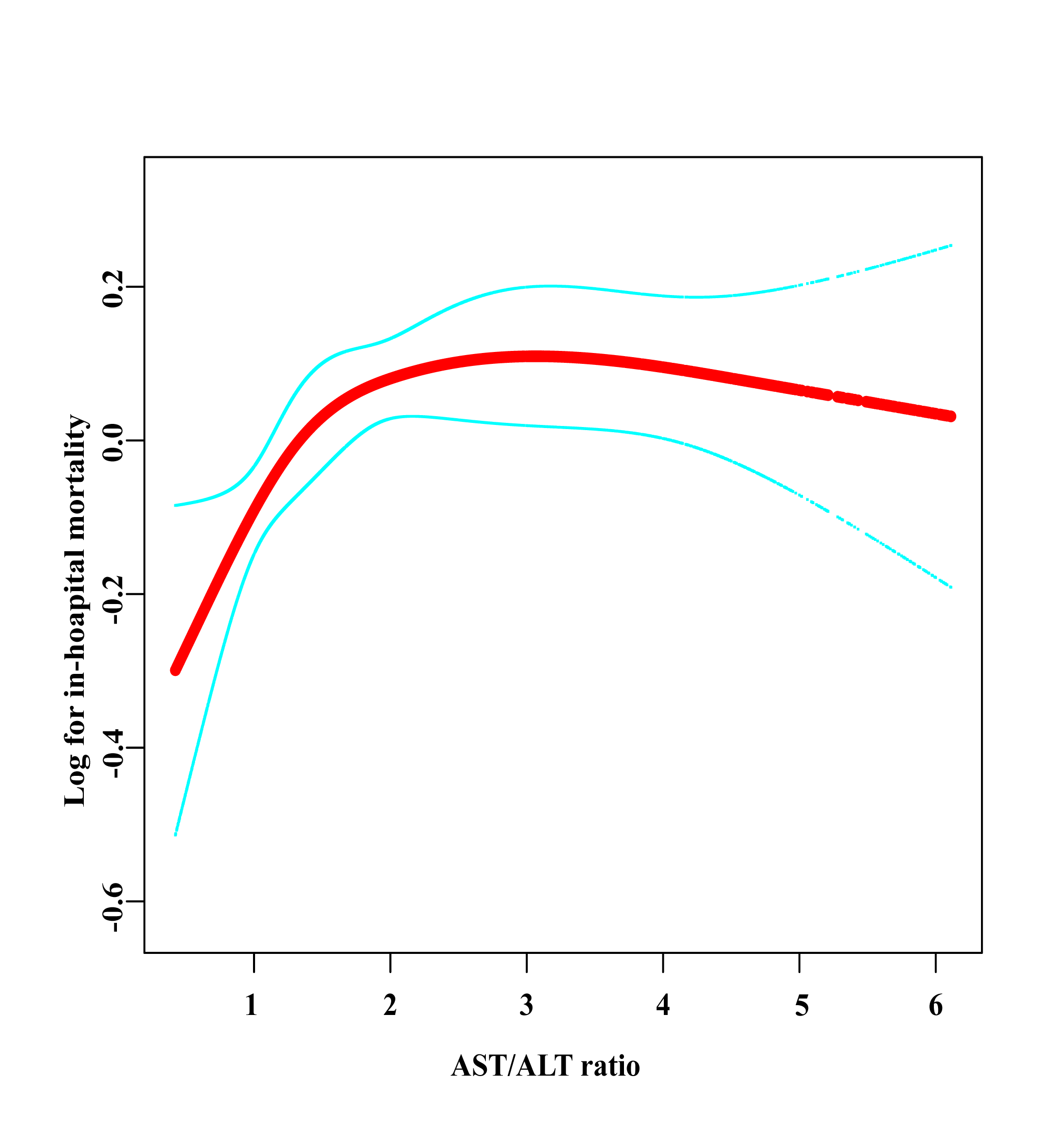

Supplement: S2 Fig — Adjusted for all factors in Model 3. The red lines represent the estimated values, and green lines represent their corresponding 95% confidence intervals. (TIF) [file pone.0293749.s005.tif]
